# Carboxythiazole is a key microbial nutrient currency and critical component of thiamin biosynthesis

**DOI:** 10.1038/s41598-018-24321-2

**Published:** 2018-04-13

**Authors:** Ryan W. Paerl, Erin M. Bertrand, Elden Rowland, Phillippe Schatt, Mohamed Mehiri, Thomas D. Niehaus, Andrew D. Hanson, Lasse Riemann, Francois-Yves Bouget

**Affiliations:** 1 Department of Marine Earth and Atmospheric Sciences, North Carolina State University, Raleigh, NC USA 27695 USA; 2 Department of Biology, University of Copenhagen, 3000 Helsingør, Denmark; 3 Department of Biology, Dalhousie University, Halifax, NS Canada; 4 Sorbonne Universités, Université Pierre and Marie Curie (Paris 06), UMR 7621, Laboratoire d’Océanographie Microbienne, Observatoire Océanologique, F-66650 Banyuls/mer, France; 5 University Nice Côte d’Azur, CNRS, Institute of Chemistry of Nice, UMR 7272, Marine Natural Products Team, Nice, France; 6 Horticultural Sciences Department, University of Florida, Gainesville, FL USA 32611 USA

## Abstract

Almost all cells require thiamin, vitamin B1 (B1), which is synthesized via the coupling of thiazole and pyrimidine precursors. Here we demonstrate that 5-(2-hydroxyethyl)-4-methyl-1,3-thiazole-2-carboxylic acid (cHET) is a useful *in vivo* B1 precursor for representatives of ubiquitous marine picoeukaryotic phytoplankton and *Escherichia coli* – drawing attention to cHET as a valuable exogenous micronutrient for microorganisms with ecological, industrial, and biomedical value. Comparative utilization experiments with the terrestrial plant *Arabidopsis thaliana* revealed that it can also use exogenous cHET, but notably, picoeukaryotic marine phytoplankton and *E. coli* were adapted to grow on low (picomolar) concentrations of exogenous cHET. Our results call for the modification of the conventional B1 biosynthesis model to incorporate cHET as a key precursor for B1 biosynthesis in two domains of life, and for consideration of cHET as a microbial micronutrient currency modulating marine primary productivity and community interactions in human gut-hosted microbiomes.

## Introduction

Thiamin (vitamin B1; called B1 herein), in the form of thiamin diphosphate, is an enzyme cofactor needed for energy generation and general metabolism in virtually all cells^1^. Despite the essentiality of B1, some populations in nature persist as B1 auxotrophs that cannot synthesize B1 *de novo* and so depend on exogenous B1 or related micronutrients to meet their B1 demands^2–4^. Cosmopolitan marine bacteria^5^, bloom-forming phytoplankton^6^, and cosmopolitan picoeukaryotic phytoplankton^6,7^, ubiquitous freshwater bacteria^8^, and about half of taxa inhabiting the human gut^9^ have been shown to be B1 auxotrophs – cumulatively highlighting the importance of exogenous B1 or related micronutrients to the operation of diverse ecosystems.

Aside from B1, precursors of B1 are also valuable exogenous micronutrients that some cells can use to meet their B1 demands^2–4,10,11^. B1 precursor use varies across prokaryotic and eukaryotic taxa^2–4^ and is thought to depend on the presence/absence of B1 biosynthesis and/or transporter genes in their respective genomes^12^. Prediction of B1 auxotrophy and/or precursor use based on gene repertoire recently helped reveal the importance of B1 precursors in sustaining environmentally significant and commercially valuable organisms. For example: (1) ubiquitous bacterioplankton, affiliated with the SAR11 clade, accounting for more than half of microbes in the oligotrophic surface ocean^13^, obligately require the pyrimidine precursor 4-amino-5-hydroxymethyl-2-methylpyrimidine (HMP) for growth^5^; (2) higher plants (*Arabidopsis thaliana* and *Zea mays*) salvage B1 from the thiazole precursor 4-methyl-5-thiazoleethanol (HET) via activity of ThiM^14^, a HET kinase previously described in model bacteria^15^; and (3) key cosmopolitan marine picoeukaryotic phytoplankton, which are significant contributors to oceanic primary production^16,17^ grow using an unidentified thiazole-related precursor(s), produced by *de novo* B1-synthesizing marine bacteria or phytoplankton, along with exogenous HMP^18^. For reference, a glossary of precursors and related enzymes referred to in this study is given in Table 1.

**Table 1:** Abbreviated names for thiamin (and its phosphorylated forms), thiamin precursor compounds, and thiamin-related enzymes referred to in the main text.

Abbreviated Name	Chemical or Enzyme Name
B1	Thiamin
TMP	Thiamin Monophosphate
TDP	Thiamin Diphosphate
HET	4-Methyl-5-thiazoleethanol
HET-P	4-Methyl-5-(2-phosphooxyethyl)thiazole
cHET	5-(2-Hydroxyethyl)-4-methyl-1,3-thiazole-2-carboxylic acid
cHET-P	4-Methyl-5-[2-(phosphonooxy)ethyl]-1,3-thiazole-2-carboxylic acid
cHET-ADP	Adenosine diphospho-5-beta-ethyl-4-methylthiazole-2-carboxylic acid
HMP	4-Amino-5-hydroxymethyl-2-methylpyrimidine
HMP-P	4-Amino-5-hydroxymethyl-2-methylpyrimidine monophosphate
HMP-PP	4-Amino-5-hydroxymethyl-2-methylpyrimidine diphosphate
ThiM	Thiazole Kinase
TMPsynthase	Thiamin Monophosphate Synthase
Thi80, TPK	Thiamin Phosphate Kinase

The picoeukaryotic phytoplankton (*Ostreococcus, Micromonas* spp.) do not grow on HET^7^, the only thiazole used in prior tests of B1 salvage from exogenous precursor(s)^1–4^. However, ThiM is required for *Ostreococcus* spp. to use the newly detected precursor(s) found in seawater and produced by *de novo* B1-synthesizing plankton - strongly suggesting that the compound(s) is thiazole-related^18^.

Intrigued that these marine picoeukaryotic phytoplankton potentially use a novel thiazole precursor, we noted with interest that carboxythiazole, 5-(2-hydroxyethyl)-4-methyl-1,3-thiazole-2-carboxylic acid (cHET), specifically phosphorylated cHET (cHET-P), is produced by bacterial thiazole synthase^19^, and that the thiazole synthase of plants and yeast similarly generates cHET-ADP (adenylation of the precursor is used rather than phosphorylation)^20,21^. Further, cHET-P is a functional substrate for bacterial thiamin monophosphate synthase (ThiE)^22^. This rigorous biochemical evidence points to cHET as a core component of *de novo* B1 biosynthesis; nonetheless, the vast majority of B1-related research and reviews to date make no mention of cHET (or phosphorylated or adenylated forms) and instead describe only synthesis and use of HET(−P)^5,7,9,23–25^. Given the apparent importance of cHET in B1 biosynthesis, and the ThiM (thiazole kinase) requirement for marine picoeukaryotic phytoplankton to use the newly detected thiazole precursor^18^, we hypothesized that cHET is a useful exogenous thiazole B1 precursor for phytoplankton.

## Results

### Marine picophytoplankton use exogenous cHET to meet their B1 demands

**Figure 1:**
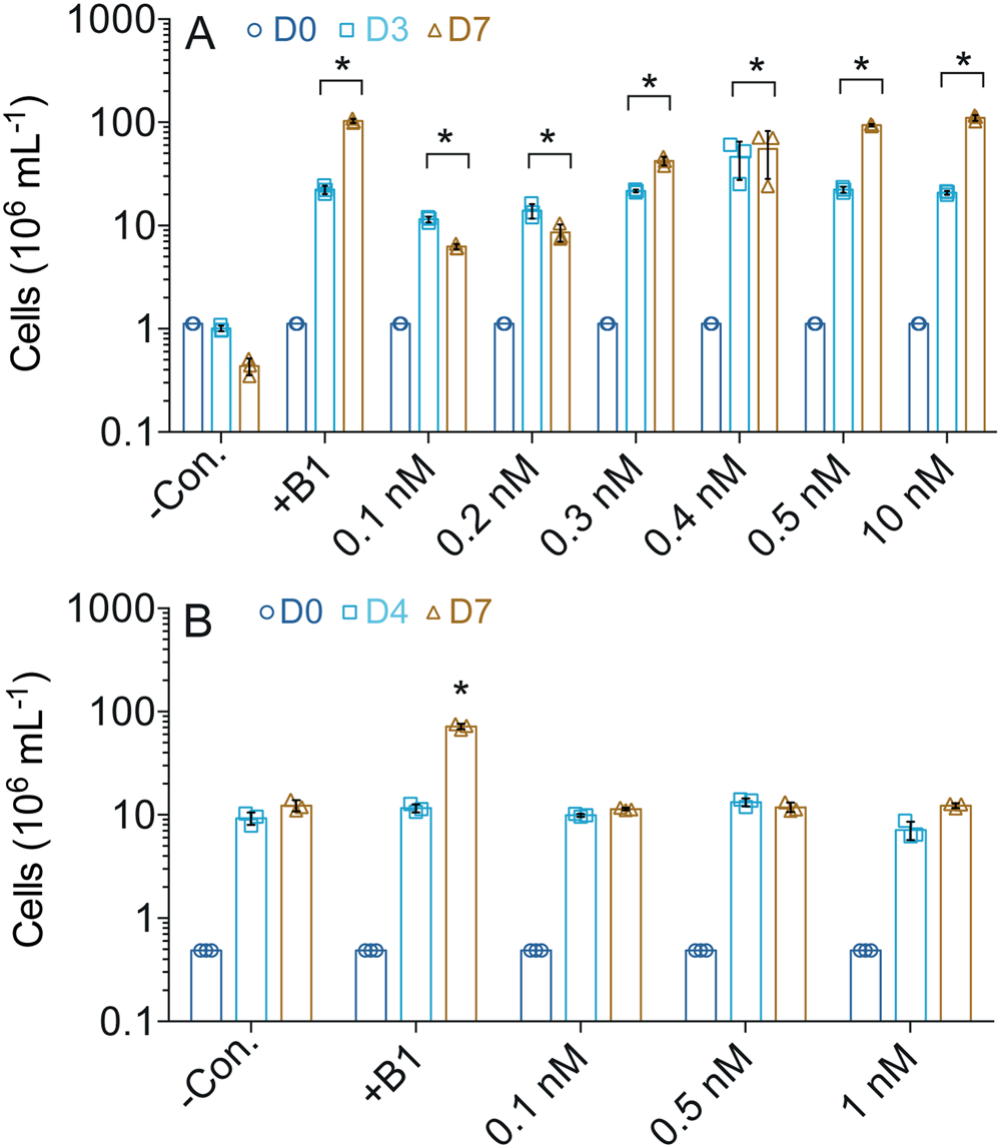
*Ostreococcus tauri* RCC745 grows on exogenous cHET (with HMP) in B1-deplete medium. Mean cell abundance data are for multiple days of the experiment (colored columns). (**A**) RCC745 grows when provided different concentrations of cHET (plus 1 nM HMP) or B1 (1 nM; as a positive control). The addition of cHET also facilitates use of low concentrations (pM) of HMP by RCC745 (Supplementary Fig. S2). (**B**) In contrast, a RCC745 ∆*thiM* mutant does not grow on supplied cHET (plus 1 nM HMP). Asterisks denote a significant difference (p < 0.05; *n* = 3; paired two-tailed t-test) relative to respective negative controls (−Con.).

In experiments with vitamin-B1 limited *Ostreococcus tauri* RCC745, a ThiM-possessing marine picoeukaryotic phytoplankton unable to use HET^18^, low additions of cHET (plus the pyrimidine precursor HMP) promoted growth (Fig. 1), confirming our hypothesis and revealing that *O. tauri* is adapted to use minute amounts of cHET dissolved in seawater. The cHET stock used in these experiments contained no detectable B1 cross-contamination, but did contain trace (0.5%) amounts of HET contamination based on selected reaction monitoring mass spectrometry (LC-SRM) (Supplementary Table S1).

Unlike wildtype *O. tauri*, a ∆*thiM* (lacking ThiM) mutant line did not grow on supplied cHET (Fig. 1B) indicating that cHET utilization requires ThiM, which agrees with prior results showing that *O. tauri* RCC745 requires ThiM in order to use the thiazole precursor produced by B1-synthesizing marine plankton^18^. Besides *O. tauri*, another cosmopolitan picoeukaryotic marine phytoplankton organism, *Bathyococcus* sp. RCC4222, also grew on supplied cHET (and HMP) under B1-limiting conditions (Supplementary Fig. S1), showing that use of exogenous cHET is a more general phenomenon in marine picoeukaryotic phytoplankton, particularly the Prasinophyceae.

### ThiM prevalence in human microbiomes and cHET use by *Escherichia coli*

Diverse organisms, including freshwater algae, enteric bacteria, human pathogens, and terrestrial plants also possess ThiM^11,14,15,18^ and hence might similarly salvage exogenous cHET for use in B1 synthesis. Bioinformatic surveys revealed that metagenomes from the human microbiome contain ~10× higher relative abundance of ThiM sequences than marine and terrestrial metagenomes (Supplementary Table S2), prompting the hypothesis that ThiM-possessing human-associated bacteria use exogenous cHET, in the same way as picoeukaryotic marine phytoplankton (Fig. 1).

**Figure 2:**
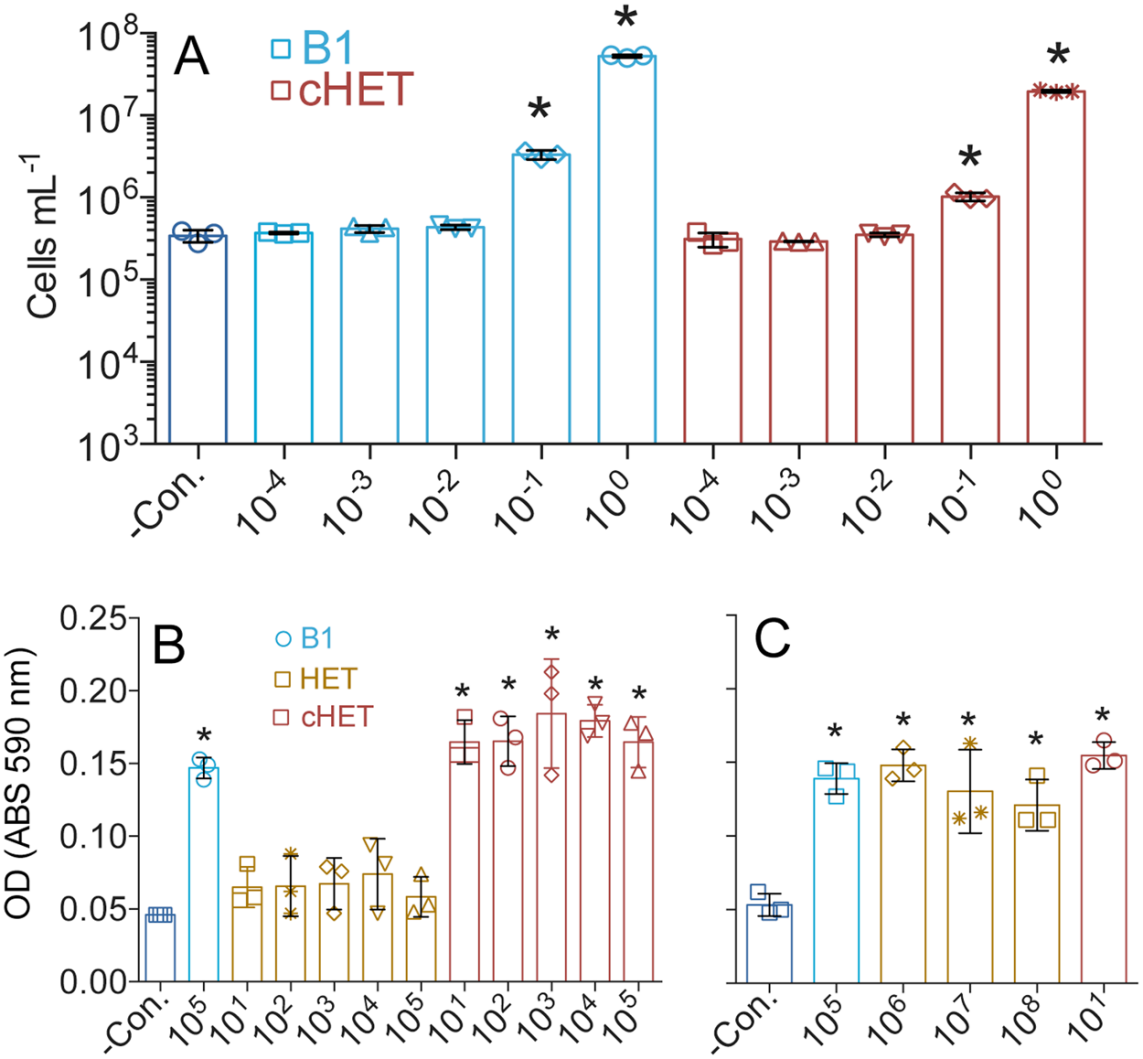
An *E. coli* ∆*thiG* mutant grows on B1-deplete M63 medium using exogenous cHET. All concentration values along the x-axes are in picomolar. (**A**) *E. coli* ∆*thiG* cells sustain growth using sub-picomolar concentrations of exogenous cHET or B1. (**B**) The *E. coli* ∆*thiG* mutant exhibited no notable growth upon supplied HET up to 10^5^ pM, highly contrasting with responses to notably lower cHET additions. (**C**) Dramatically higher concentrations (≥10^6^ pM) of exogenous HET are required to sustain growth of *E. coli* ∆*thiG*. Mean maximum yields for triplicate cultures are plotted along with their respective standard deviations. Asterisks denote significant differences (*p* < 0.05, *n* = 3; paired two-tailed t-test) versus the negative control (−Con.).

Human-associated enteric bacterium *Escherichia coli* K-12 as well as >400 other *E. coli* strains possess ThiM^15^ (Supplementary Table S3), making *Escherichia coli* a suitable model for testing our hypothesis. Experiments with an *E. coli* mutant lacking ThiG (∆*thiG*), the enzyme that synthesizes the thiazole precursor of B1 in *de novo* biosynthesis, showed the bacterium is also adapted to use low concentrations of exogenous cHET, specifically down to subpicomolar concentrations (Fig. 2). In contrast, ~1 million times more (>100 nM) HET was necessary to support comparable growth (Fig. 2), which confirms our hypothesis and is also consistent with prior experiments indicating that micromolar concentrations of HET are needed to sustain *E. coli* growth^15^. No trace cHET contamination was detected in HET stocks (Supplementary Table S1), suggesting that *E. coli* can synthesize B1 from HET, but only at relatively high extracellular concentrations of HET, which presumably enters the cell via low-affinity transporters and/or diffusion.

### cHET use by the terrestrial plant Arabidopsis

**Figure 3:**
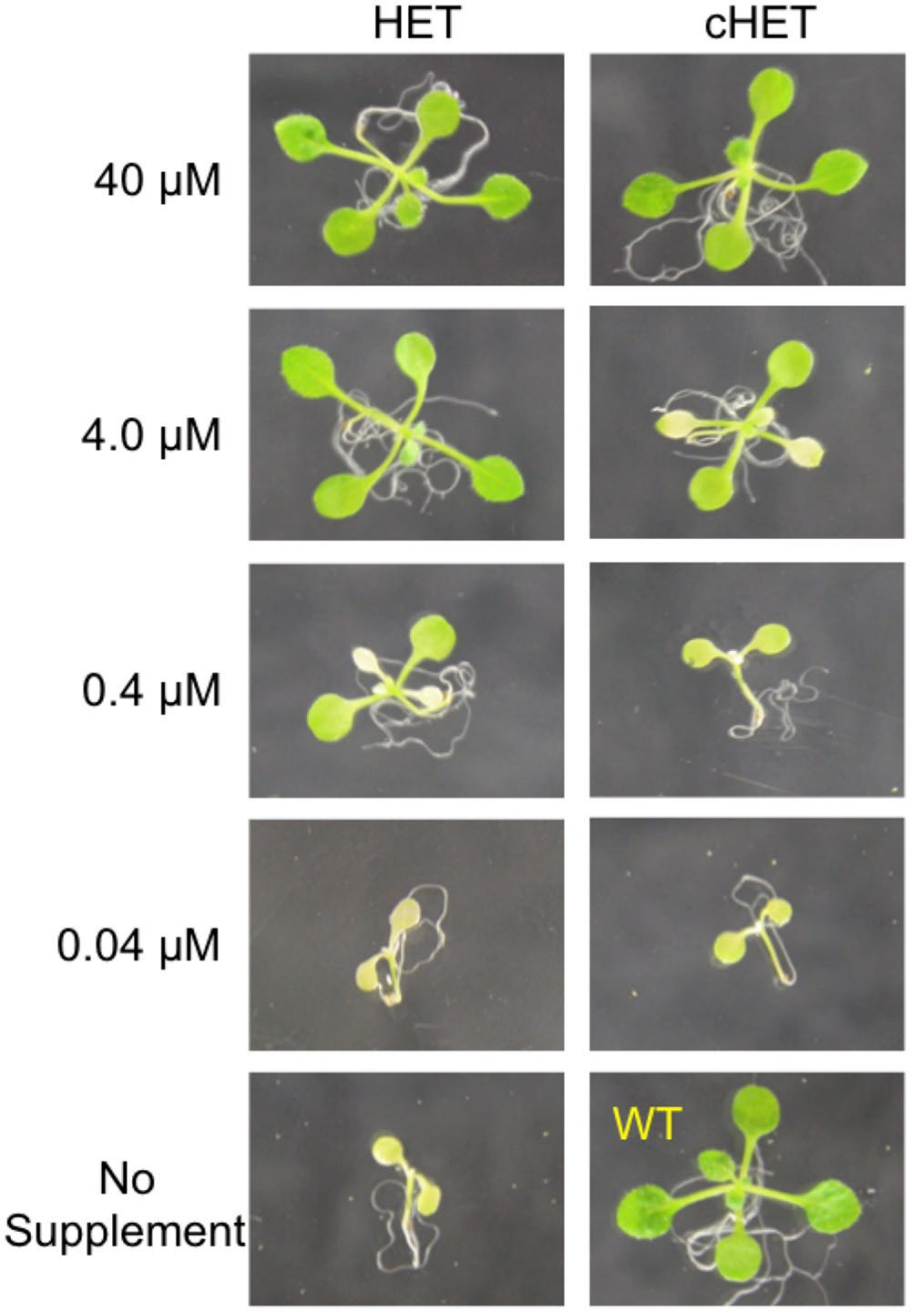
Thiazole-auxotrophic *Arabidopsis thaliana* plants can use cHET to sustain growth. Mutant *tz-1* plants were grown with the indicated concentrations of HET or cHET. Pictures were taken 14 days after germination and are representative of at least 30 plants. A wild-type (WT) plant is shown for comparison.

Broadly contextualizing microbial use of cHET, we also tested whether ThiM-possessing terrestrial plants such as *Arabidopsis*^14^ can use exogenous cHET. Growth experiments with *Arabidopsis*, using the wild-type and a mutant (*tz-1*) unable to synthesize thiazole precursor, confirmed that *Arabidopsis* can use cHET because the mutant grew well when given high concentrations of cHET (Fig. 3). In contrast to picoeukaryotic phytoplankton and *E. coli* (Figs 1, 2), equivalent concentrations of HET also sustained growth of the *Arabidopsis* mutant (Fig. 3). This result highlights a key difference between plants and (aquatic) microbes, in that the latter are equipped to salvage B1 from very low concentrations of exogenous precursors (Figs 1, 2), likely as a result of adaption within an environment where precursor(s) are a community currency circulated between producers and consumers.

## Discussion

Collectively, our findings establish cHET as a valuable microbial B1-related currency and component of *de novo* B1 biosynthesis. Previously, HET was the only thiazole considered in research investigating B1 salvage^1–4,7,9,14,25^. Our results alter this paradigm as cHET is clearly useful for prokaryotic and eukaryotic microorganisms, and moreover is accessible at extremely low concentrations (Figs 1, 2). Specifically, acquisition of exogenous cHET sustains key primary producers in the ocean as well as important enterobacteria, presumably enabling them to bypass the energetic and/or elemental costs of *de novo* biosynthesis of cHET(−P). The ability of these cells, which are endemic to marine euphotic waters and the human body, to use very low exogenous cHET concentrations strongly suggests that cHET is bioavailable in nature and integral to interactions between B1 auxotrophs and B1-synthesizing microorganisms or hosts. However, direct evidence of this is lacking.

**Figure 4:**
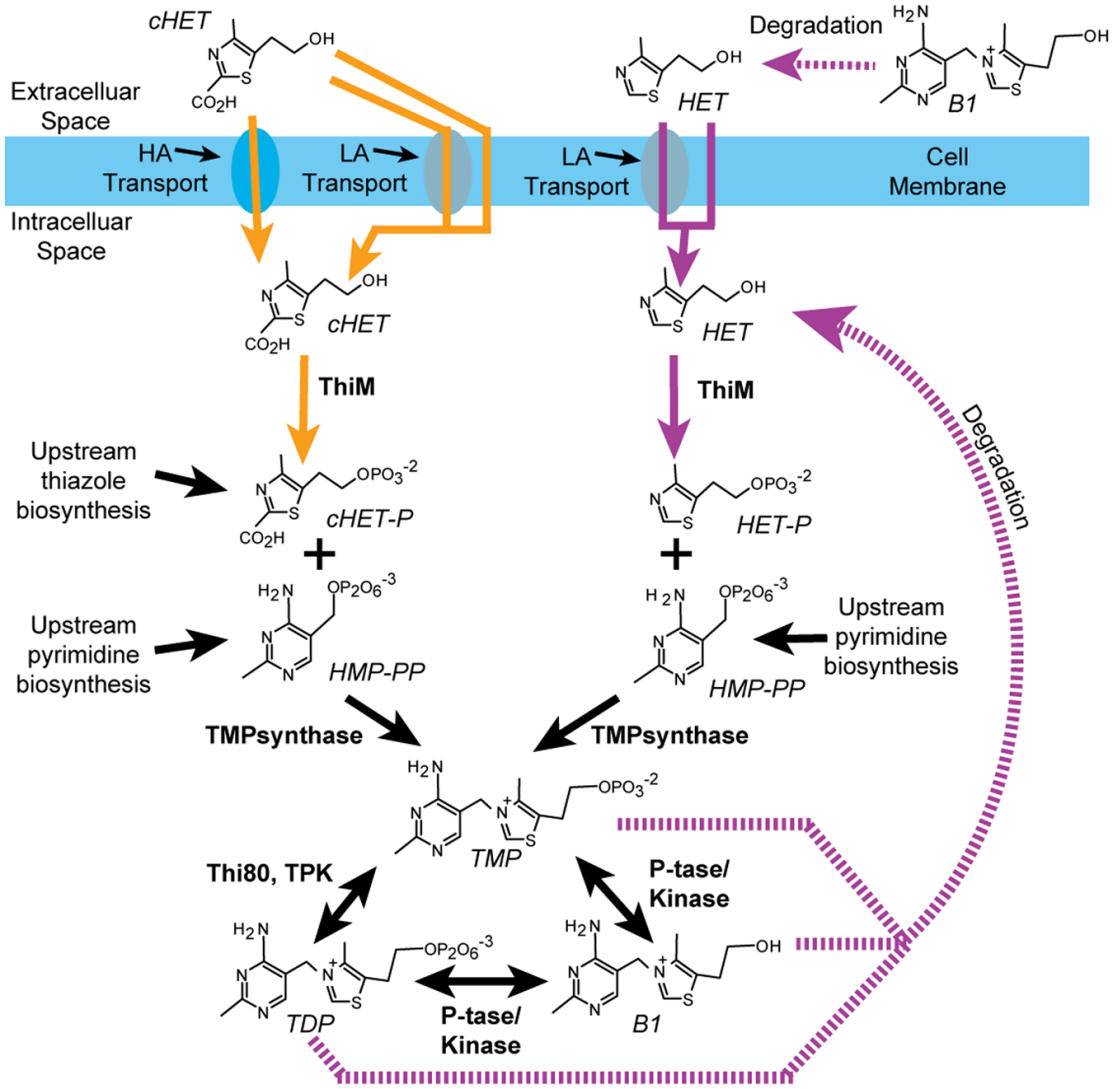
An updated metabolic map including cHET in B1 salvage and *de novo* biosynthesis pathways. Orange arrows represent cHET utilization demonstrated here. Solid and dashed purple arrows represent usage and generation (via degradation) of HET reported previously^1,2,4,30^. Large black arrows denote core B1 biosynthesis processes. Cells acquire exogenous thiazole precursor via high (HA) or low affinity (LA) transport systems (with unknown sequence identity), or diffusion based on results shown here (Figs 1, 2, Supplementary Fig. S1) and in prior studies^4,30^. Shorthand compound names are in italics, while shorthand enzyme names are in bold. Thi80, TPK = thiamin pyrophosphate kinase; TMPsynthase = thiamin monophosphate synthase; P-tase = phosphatase; TMP = thiamin monophosphate; TDP = thiamin diphosphate; B1 = thiamin; −P = phosphate group; HMP = 4-amino-5-hydroxymethyl-2-methylpyrimidine; cHET = 5-(2-hydroxyethyl)-4-methyl-1,3-thiazole-2-carboxylic acid; HET = 4-methyl-5-thiazoleethanol.

Our observation that diverse organisms grow on cHET (Figs 1–3, Supplementary Fig. S1), alongside prior biochemical evidence of cHET-P generation by bacteria and plants^19,21^, solidifies cHET-P as a fundamental and widely overlooked^5,7,9,14,23–25^ component of *de novo* B1 biosynthesis (Fig. 4). Establishing that cHET is central to *de novo* B1 biosynthesis pinpoints B1-prototrophic organisms as sources of the thiazole to co-occurring populations, i.e. key microorganisms that influence marine primary productivity (Fig. 1) or human health (e.g. Shiga-toxin producing *E. coli* (STEC)^26^ possessing ThiM, e.g. *E. coli* STEC_O31; Supplementary Table S3). Our findings also improve understanding of B1 biosynthesis in general - a vital process for life on Earth, and target for industrial and biomedical applications with human impact, e.g. efforts to increase crop nutritive value or resilience^27,28^ and to develop drugs targeting pathogenic microbes^29^.

For clarity, we propose that HET be regarded as a degradation-derived precursor as it comes from B1 degradation^1,30^, whereas cHET is generated via the *de novo* biosynthesis pathway^19–21^ (Fig. 4). Comparable descriptors also apply to pyrimidine B1 precursors 4-amino-5-aminomethyl-2-methylpyrimidine (AmMP; degradation product) and HMP (product of *de novo* biosynthesis)^1,10,30^.

In conclusion, prevailing views of thiazole precursor biosynthesis, use, and exchange require reassessment, as it is now evident that the role of B1 requirements in host-associated or aquatic microbiomes cannot be fully understood without consideration of the widely overlooked thiazole B1 precursor cHET(−P). Exchange of cHET and its influence upon interactions between taxa^31^, especially producers and consumers of cHET, deserves particular attention given that exogenous cHET acquisition is integral to the survival of microorganisms with environmental, industrial, and/or biomedical impacts, e.g. key marine microbial primary producers, *E. coli*, and other ThiM-possessing taxa^7^ (Figs 1, 2; Supplementary Table S2). Since model organisms readily utilize exogenous cHET, it should be possible for future research to decipher the intricacies of cHET flux and its influence upon cell interactions.

## Methods

### Chemicals

Thiamin hydrochloride and 4-methyl-5-thiazoleethanol (HET) (≥95% HPLC-determined purity) were purchased from Sigma Aldrich (St. Louis, MO, USA), and 4-amino-5-hydroxymethyl-2-methylpyrimidine (HMP) (>95% purity) was purchased from Enamine Ltd. (Kiev, Ukraine). 5-(2-Hydroxyethyl)-4-methyl-1,3-thiazole-2-carboxylic acid (cHET) (>98% HPLC-determined purity) was purchased from Finetech Industry Limited (Wuhan, China).

### Marine phytoplankton growth experiments

Cultures of *O. tauri* RCC745 wild-type, the ∆*thiM* mutant line, and *Bathycoccus* sp. RCC4222 were maintained at 20 °C under 25 μE m^−2^ s^−1^ white light on artificial seawater (ASW) supplemented with trace metals and B1 and B12 vitamins as previously described^18,32^. Antibiotics (penicillin G 50 μg mL^−1^, streptomycin 200 μg ml^−1^) were added to prevent the growth of *Marinobacter* bacteria associated with *O. tauri* RCC745 cultures^18^. RCC745 cell growth and the absence of *Marinobacter* were determined on an Accuri C6 flow cytometer (Becton Dickinson) using SYBR Green Il staining. To start cHET bioassays, algal cells inoculated at 0.5 to 1 × 10^6^ cells mL^−1^ were first grown for 7 days in B1-deplete ASW medium (ASW-B1). These B1-deprived cultures were used to inoculate ASW culture medium containing HMP (1 nM) and various concentrations of cHET at 0.5 to 1 × 10^6^ cells mL^−1^ in triplicate. Similar experiments were performed to determine the requirements for B1 or HMP (in 1 nM cHET-supplemented ASW). Triplicate positive (1 nM B1) and negative (ASW-B1) controls were run in parallel. Microalgal cell abundances were determined on an Accuri C6 flow cytometer.

### Escherichia coli growth experiments

*E. coli* JW5549 Δ*thiG*761::kan (Keio Collection) cells were cultured in LB medium. Cells were harvested via centrifugation (9 × 1000 g; 3 min), washed, and resuspended over three iterations in M63 minimal growth medium^33^ lacking B1 and only 1 mM glucose as a carbon source to minimize the potential for vitamin contamination from glucose stock. Washed/resuspended cells were added to triplicate sterile 4.5 mL polystyrene tubes containing M63 B1-free test medium (at a ratio of 0.5 µL cells: 1.5 mL medium) with various concentrations of HET or cHET, B1 (as a positive control), or without amendment (as a negative control). Static culture tubes were incubated at room temperature, in the dark. Cultures were thoroughly vortexed before sampling after one and two weeks of incubation. Optical density (590 nm) of cultures was measured using a FLUOstar Optima Plate Reader (Bmg-Labtech) and clear 96-well plates. Cell abundances were determined from fixed (2% formaldehyde) and frozen (−80 °C) culture samples via SYBR green I (Molecular Probes, Eugene, OR, USA) staining and flow cytometry^34^ using a FACS CANTO II (Beckton Dickenson, Heidelberg, Germany).

### Plant growth experiments

*Arabidopsis thaliana* thiazole-auxotrophic mutant (*tz-1*; ABRC stock number CS3375)^35^ and wild type (Columbia-1; CS3176) seeds were surface sterilized and plated on ½ MS medium containing 0.6% (w/v) Phytagel, 1% (w/v) sucrose, and with or without various concentrations of HET or cHET. Plates were held in the dark at 4 °C for four days, then placed under fluorescent lights (130–150 µE m^−1^ s^−1^) on a 12:12 h light/dark cycle at 22 °C for 14 days.

### Liquid chromatography-mass spectrometry

Sample preparation: Stock solutions (1 mg mL^−1^) and intermediate stock solutions (10 µg mL^−1^) of HET, cHET, HMP, and B1 were prepared in Milli Q water and stored at −20 °C in the dark. Working solutions were prepared as 0.02 µg µL^−1^ solutions in 5 mM ammonium formate, 0.1% formic acid and 10% methanol for LC-SRM analysis.

LC-SRM: Working stocks of all four compounds were prepared in 5 mM ammonium formate, 0.1% formic acid and 10% methanol. One microliter injections onto a 150 × 0.3 mm ID column (Acclaim PepMap RSLC, C-18, 2 µm, 100 Å), held at 50 degrees C and subject to an HPLC gradient of 2–6% B over 4 min, then 6 to 10% B over 1.5 min (A, 0.1% formic acid; B, 80% acetonitrile, 0.08% formic acid) at 7 µl per min. This was coupled to a Thermo Quantiva triple quadrupole mass spectrometer in selected reaction monitoring (SRM) mode, operating under the following conditions: Q1 and Q3 resolution 0.4 (FWHM), 50 msec dwell time, spray voltage 3500 (positive ion mode), sheath gas 6, aux gas 2, ion transfer tube 235 C, vaporizer temp 70 C°. SRM parameters for each compound are given in Table S4. Limits of quantitation and limits of detection were calculated as described previosuly^36^ using a standard curve created from repeat injections of 0, 10, 100, 200, 500 and 1000 fmol of each compound on column and are given in Table S3. Cross contamination was assessed and reported for triplicate injections of 1000 fmol of each compound on column.

### Metagenomic queries

The *E. coli* ThiM amino acid sequence (Uniprot ID P76423) was searched against diverse metagenomic libraries using BLASTP (and default BLOSUM62 settings) via GenomeNet (http://www.genome.jp/tools/blast/; Kyoto University Bioinformatics Center). Queries of soil metagenomic libraries were performed using BLASTP searches via the Joint Genome Institute (JGI) Integrated Microbial Genome (IMG) portal^37^.

### Data availability statement

Data sharing is not applicable to this article as no datasets were generated or analyzed during the current study.

## Electronic supplementary material

Supplementary Information
