# Author Correction: Carboxythiazole is a key microbial nutrient currency and critical component of thiamin biosynthesis

**DOI:** 10.1038/s41598-018-27042-8

**Published:** 2018-06-06

**Authors:** Ryan W. Paerl, Erin M. Bertrand, Elden Rowland, Phillippe Schatt, Mohamed Mehiri, Thomas D. Niehaus, Andrew D. Hanson, Lasse Riemann, Francois-Yves Bouget

**Affiliations:** 1 Department of Marine Earth and Atmospheric Sciences, North Carolina State University, Raleigh, NC USA 27695 USA; 2 Department of Biology, University of Copenhagen, 3000 Helsingør, Denmark; 3 Department of Biology, Dalhousie University, Halifax, NS Canada; 4 Sorbonne Universités, Université Pierre and Marie Curie (Paris 06), UMR 7621, Laboratoire d’Océanographie Microbienne, Observatoire Océanologique, F-66650 Banyuls/mer, France; 5 University Nice Côte d’Azur, CNRS, Institute of Chemistry of Nice, UMR 7272, Marine Natural Products Team, Nice, France; 6 Horticultural Sciences Department, University of Florida, Gainesville, FL USA 32611 USA

Correction to: *Scientific Reports* 10.1038/s41598-018-24321-2, published online 13 April 2018

The original version of this Article contained a typographical error in the spelling of the author Francois-Yves Bouget, which was incorrectly given as Francois Yves-Bouget. This has now been corrected in the PDF and HTML versions of the Article and in the accompanying Supplementary Information file.

