# Supplementary material for: Carboxythiazole is a key microbial nutrient currency and critical component of thiamin biosynthesis
Source: Sci Rep. 2018 Apr 13;8:5940. doi: 10.1038/s41598-018-24321-2 (PMC5899164; doi:10.1038/s41598-018-24321-2)
Supplement: Supplementary file 1 — Supplementary Information [file 41598_2018_24321_MOESM1_ESM.docx]

**Supplementary Information for:**

**Carboxythiazole is a key microbial nutrient currency and critical component of thiamin biosynthesis**

Ryan W. Paerl; Erin M. Bertrand; Elden Rowland; Phillippe Schatt, Mohamed Mehiri; Thomas D. Niehaus; Andrew D. Hanson; Lasse Riemann; Francois-Yves Bouget

Supplementary Figure S1. *Bathycoccus* sp. RCC4222 grows in B1-deplete medium upon supplied cHET (plus HMP, 1 nM). Means (columns) and standard deviations (error bars) are from triplicate cultures. The positive control (+B1) received 1 nM B1, and the negative control (-B1) no addition.


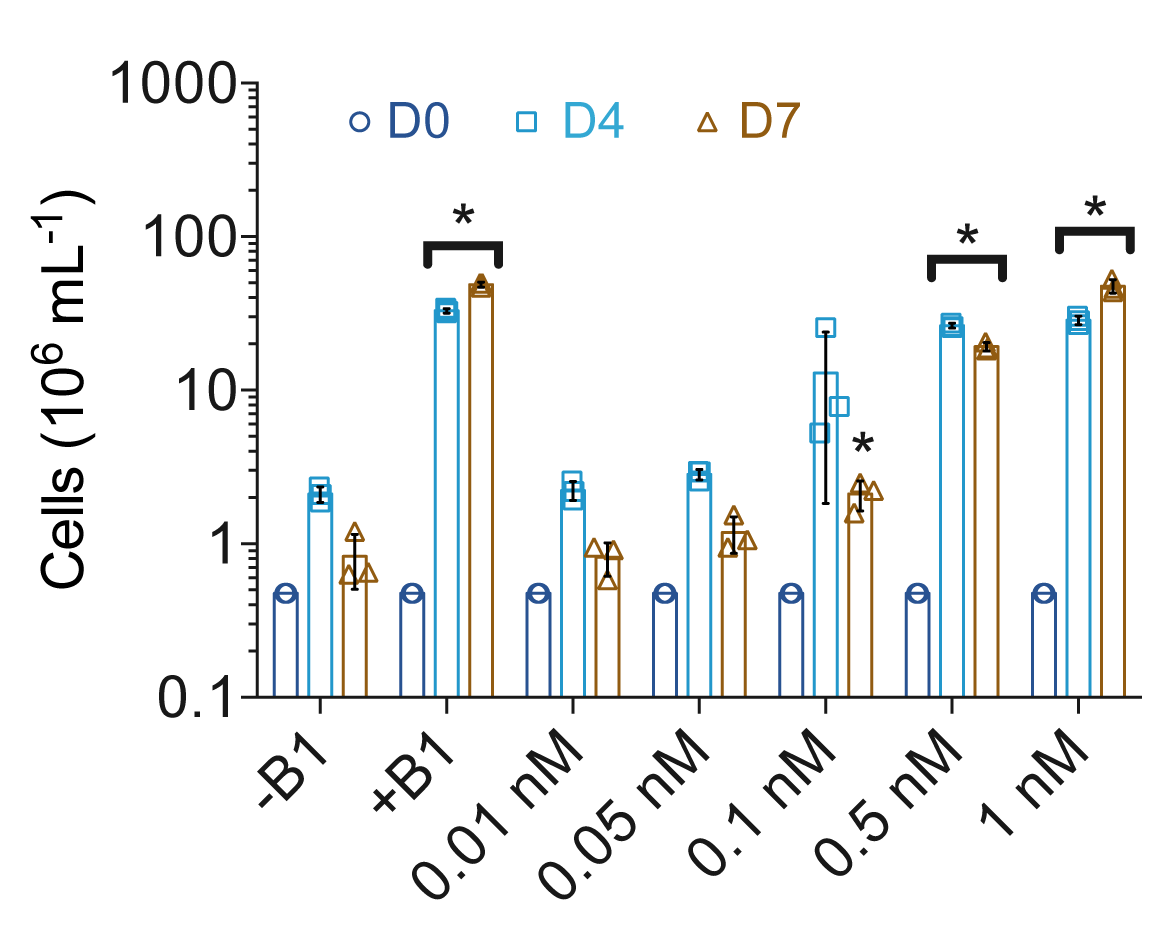


Supplementary Figure S2. Increasing growth of B1-limited *Ostreococcus tauri* RCC745 with increasing addition of exogenous B1 (A) or HMP (B) (plus 1 nM cHET). The plotted data represent means (columns) and standard deviations (error bars) for triplicate cultures.


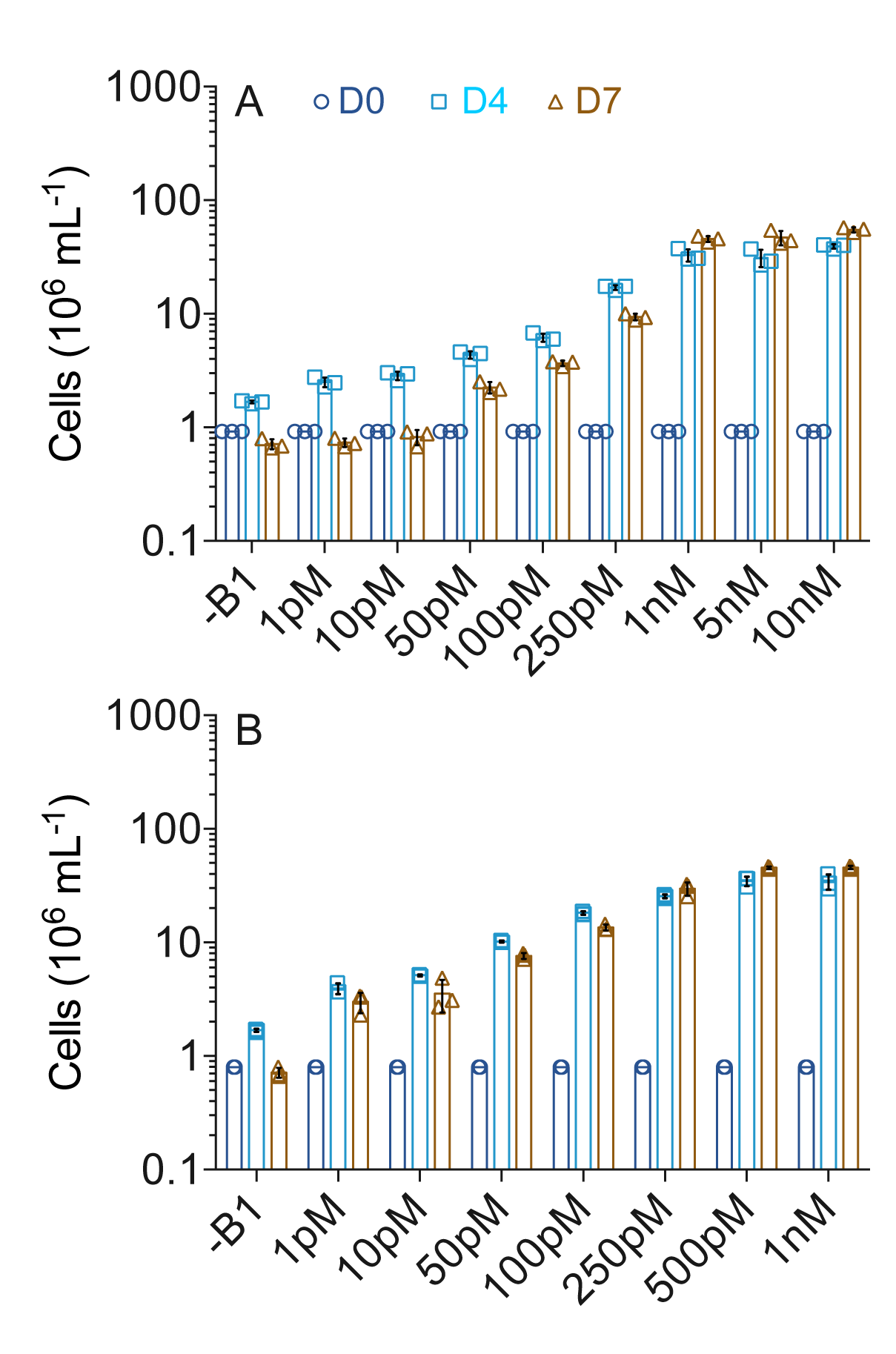


Supplementary Table S1. Cross contamination matrix, including limits of detection (LOD) and limits of quantitation (LOQ), denoting when HET, cHET, HMP and B1 were detected and quantified in 1000 fmol injections commercially available sources of HET, cHET, HMP and B1 via LC-SRM-MS. nd = < LOD; nq = < LOQ.

|  | **Cross Contamination (fmol)** | | | |
| --- | --- | --- | --- | --- |
| **Compound Injected** | **HET** | **cHET** | **HMP** | **B1** |
| **HET** |  | nd | nd | nd |
| **cHET** | 5 |  | nd | nd |
| **HMP** | nd | nd |  | nd |
| **B1** | nd | nd | nq |  |
|  |  |  |  |  |
| **LOD (fmol)** | 1 | 3 | 7 | 5 |
| **LOQ (fmol)** | 2 | 5 | 9 | 8 |

Supplementary Table S2. The percent abundance of ThiM sequences varies across marine, human microbiome, and soil metagenomic libraries. Human microbiome derived sequence libraries contained the highest relative abundance of ThiM sequences (highlighted in light blue). * = 21 sequences with closest similarity to ADP-dependent NAD(P)H-hydrate dehydratase were manually removed from the total.

| **Source** | **Sample** | **KEGG or IMG Genome ID** | **Positive Hits** | **Total Sequences** | **Percent Positive Hits** |
| --- | --- | --- | --- | --- | --- |
| Ocean | Global Ocean Survey (GOS) | CAMERA/GOS | 215 | 4.11E+07 | 0.0005% |
| Ocean | Tara Oceans | Tara Oceans | 978* | 1.12E+08 | 0.0009% |
| Ocean | Ocean Science Day (OSD) | OSD | 67 | 7.02E+06 | 0.0010% |
| Human Microbiome | Human Gut | Metagenome.jp (T30003 to T30015) | 93 | 6.63E+05 | 0.0140% |
| Human Microbiome | Human Gut | MetaHIT (T30016 to T30040) | 278 | 2.32E+06 | 0.0120% |
| Human Microbiome | Human Gut | MetaHIT (T30041 to T30080) | 437 | 4.24E+06 | 0.0103% |
| Human Microbiome | Human Gut | MetaHIT (T30081 to T30120) | 531 | 4.14E+06 | 0.0128% |
| Human Microbiome | Human Gut | MetaHIT (T30121 to T30139) | 313 | 2.13E+06 | 0.0147% |
| Human Microbiome | Human Skin | HMP - Skin (Category) | 145 | 6.42E+05 | 0.0226% |
| Human Microbiome | Human Urinary/Genital Organs | HMP - Urogenital (Category) | 15 | 6.45E+04 | 0.0232% |
| Human Microbiome | Human Airway | HMP - Airway (Category) | 92 | 3.78E+05 | 0.0244% |
| Human Microbiome | Human Buccal Mucosa | HMP - Oral (Category) | 483 | 3.19E+06 | 0.0152% |
| Soil | Grasslands | 3300001305 | 30 | 1.96E+06 | 0.0015% |
| Soil | Grasslands | 3300001686 | 86 | 5.27E+06 | 0.0016% |
| Soil | Grasslands | 3300002568 | 291 | 2.31E+07 | 0.0013% |
| Soil | Grasslands | 3300001205 | 11 | 6.84E+05 | 0.0016% |
| Soil | Hubbard Brook Forest | 3300005591 | 25 | 7.55E+06 | 0.0003% |
| Soil | Hubbard Brook Forest | 3300005602 | 53 | 8.81E+06 | 0.0006% |
| Soil | Peat Soil | 3300001356 | 32 | 2.00E+06 | 0.0016% |
| Soil | Peat Soil | 3300000567 | 15 | 1.53E+06 | 0.0010% |

Supplementary Table S3. *Escherichia coli* strains (454, potentially more considering multispecies hits) that possess ThiM based on NCBI BLASTP searches using UNIPROT *E. coli* K12 ThiM sequence P76423. All positive BLASTP hits exhibited E values of 0.0 and bit scores of ≥525.

| **Strain** | **Number of hits** |
| --- | --- |
| *Escherichia coli 'BL21-Gold(DE3)pLysS AG'* | 1 |
| *Escherichia coli 1-110-08_S1_C3* | 1 |
| *Escherichia coli 1-110-08_S4_C2* | 1 |
| *Escherichia coli 1-176-05_S1_C2* | 1 |
| *Escherichia coli 1-176-05_S1_C3* | 1 |
| *Escherichia coli 1-176-05_S4_C1* | 1 |
| *Escherichia coli 1-176-05_S4_C2* | 1 |
| *Escherichia coli 1-176-05_S4_C3* | 1 |
| *Escherichia coli 1-182-04_S3_C1* | 1 |
| *Escherichia coli 1-182-04_S3_C2* | 1 |
| *Escherichia coli 1-182-04_S3_C3* | 1 |
| *Escherichia coli 1-250-04_S3_C1* | 1 |
| *Escherichia coli 1-250-04_S3_C2* | 1 |
| *Escherichia coli 101-1* | 1 |
| *Escherichia coli 113302* | 1 |
| *Escherichia coli 1303* | 1 |
| *Escherichia coli 2-005-03_S1_C2* | 1 |
| *Escherichia coli 2-005-03_S1_C3* | 1 |
| *Escherichia coli 2-005-03_S4_C1* | 1 |
| *Escherichia coli 2-011-08_S1_C3* | 1 |
| *Escherichia coli 2-011-08_S3_C1* | 1 |
| *Escherichia coli 2-011-08_S3_C2* | 1 |
| *Escherichia coli 2-011-08_S4_C1* | 1 |
| *Escherichia coli 2-011-08_S4_C3* | 1 |
| *Escherichia coli 2-052-05_S1_C1* | 1 |
| *Escherichia coli 2-052-05_S1_C3* | 1 |
| *Escherichia coli 2-052-05_S3_C1* | 1 |
| *Escherichia coli 2-052-05_S3_C2* | 1 |
| *Escherichia coli 2-052-05_S3_C3* | 1 |
| *Escherichia coli 2-156-04_S4_C3* | 1 |
| *Escherichia coli 2-177-06_S1_C3* | 1 |
| *Escherichia coli 2-177-06_S3_C1* | 1 |
| *Escherichia coli 2-210-07_S3_C1* | 1 |
| *Escherichia coli 2-210-07_S4_C1* | 1 |
| *Escherichia coli 2-210-07_S4_C2* | 1 |
| *Escherichia coli 2-210-07_S4_C3* | 1 |
| *Escherichia coli 2-222-05_S1_C2* | 1 |
| *Escherichia coli 2-222-05_S1_C3* | 1 |
| *Escherichia coli 2-222-05_S4_C1* | 1 |
| *Escherichia coli 2-222-05_S4_C2* | 1 |
| *Escherichia coli 2-222-05_S4_C3* | 1 |
| *Escherichia coli 2-316-03_S3_C1* | 1 |
| *Escherichia coli 2-316-03_S4_C2* | 1 |
| *Escherichia coli 2-460-02_S4_C1* | 1 |
| *Escherichia coli 2-474-04_S1_C2* | 1 |
| *Escherichia coli 2-474-04_S3_C1* | 1 |
| *Escherichia coli 2-474-04_S3_C2* | 1 |
| *Escherichia coli 2-474-04_S3_C3* | 1 |
| *Escherichia coli 201600.1* | 1 |
| *Escherichia coli 2731150* | 1 |
| *Escherichia coli 2861200* | 1 |
| *Escherichia coli 2872000* | 1 |
| *Escherichia coli 3-020-07_S1_C1* | 1 |
| *Escherichia coli 3-020-07_S1_C2* | 1 |
| *Escherichia coli 3-020-07_S1_C3* | 1 |
| *Escherichia coli 3-020-07_S3_C1* | 1 |
| *Escherichia coli 3-020-07_S3_C2* | 3 |
| *Escherichia coli 3-073-06_S1_C2* | 1 |
| *Escherichia coli 3-073-06_S3_C1* | 1 |
| *Escherichia coli 3-073-06_S3_C2* | 1 |
| *Escherichia coli 3-073-06_S4_C1* | 1 |
| *Escherichia coli 3-073-06_S4_C3* | 1 |
| *Escherichia coli 3-105-05_S1_C2* | 1 |
| *Escherichia coli 3-105-05_S3_C1* | 1 |
| *Escherichia coli 3-105-05_S3_C2* | 1 |
| *Escherichia coli 3-105-05_S3_C3* | 1 |
| *Escherichia coli 3-267-03_S3_C1* | 1 |
| *Escherichia coli 3-373-03_S1_C2* | 1 |
| *Escherichia coli 3-373-03_S1_C3* | 1 |
| *Escherichia coli 3-373-03_S3_C1* | 1 |
| *Escherichia coli 3-373-03_S4_C1* | 1 |
| *Escherichia coli 3-373-03_S4_C2* | 1 |
| *Escherichia coli 3-373-03_S4_C3* | 1 |
| *Escherichia coli 3-475-03_S3_C2* | 1 |
| *Escherichia coli 3.2608* | 1 |
| *Escherichia coli 4-203-08_S1_C1* | 1 |
| *Escherichia coli 4-203-08_S1_C2* | 1 |
| *Escherichia coli 4-203-08_S1_C3* | 1 |
| *Escherichia coli 4-203-08_S4_C2* | 1 |
| *Escherichia coli 5-172-05_S1_C3* | 1 |
| *Escherichia coli 5-172-05_S3_C1* | 1 |
| *Escherichia coli 5-172-05_S4_C1* | 1 |
| *Escherichia coli 5-172-05_S4_C2* | 1 |
| *Escherichia coli 5-172-05_S4_C3* | 1 |
| *Escherichia coli 5-366-08_S4_C2* | 1 |
| *Escherichia coli 5.0959* | 1 |
| *Escherichia coli 541-15* | 1 |
| *Escherichia coli 55989* | 2 |
| *Escherichia coli 6-319-05_S4_C2* | 1 |
| *Escherichia coli 7-233-03_S1_C2* | 1 |
| *Escherichia coli 7-233-03_S3_C3* | 1 |
| *Escherichia coli 7-233-03_S4_C1* | 1 |
| *Escherichia coli 7-233-03_S4_C2* | 1 |
| *Escherichia coli 7-233-03_S4_C3* | 1 |
| *Escherichia coli 8-415-05_S1_C1* | 1 |
| *Escherichia coli 8-415-05_S1_C2* | 1 |
| *Escherichia coli 9.0111* | 1 |
| *Escherichia coli 907710* | 1 |
| *Escherichia coli 908541* | 1 |
| *Escherichia coli 909945-2* | 1 |
| *Escherichia coli 96.0497* | 1 |
| *Escherichia coli ACN001* | 1 |
| *Escherichia coli AD30* | 1 |
| *Escherichia coli APEC O78* | 1 |
| *Escherichia coli ATCC 35150* | 1 |
| *Escherichia coli ATCC 8739* | 2 |
| *Escherichia coli ATCC BAA-2215* | 1 |
| *Escherichia coli ATCC BAA-2219* | 1 |
| *Escherichia coli B* | 2 |
| *Escherichia coli B str. REL606* | 1 |
| *Escherichia coli B088* | 1 |
| *Escherichia coli B171* | 1 |
| *Escherichia coli B41* | 1 |
| *Escherichia coli B7A* | 1 |
| *Escherichia coli B921* | 1 |
| *Escherichia coli BCE006_MS-23* | 1 |
| *Escherichia coli BCE007_MS-11* | 1 |
| *Escherichia coli BCE008_MS-01* | 1 |
| *Escherichia coli BCE008_MS-13* | 1 |
| *Escherichia coli BCE019_MS-13* | 1 |
| *Escherichia coli BCE030_MS-09* | 1 |
| *Escherichia coli BCE032_MS-12* | 1 |
| *Escherichia coli BIDMC 37* | 1 |
| *Escherichia coli BIDMC 59* | 1 |
| *Escherichia coli BL21(DE3)* | 2 |
| *Escherichia coli BW25113* | 1 |
| *Escherichia coli BW2952* | 2 |
| *Escherichia coli C* | 1 |
| *Escherichia coli C-34666* | 1 |
| *Escherichia coli C691-71 (14b)* | 1 |
| *Escherichia coli CE418* | 1 |
| *Escherichia coli CE516* | 1 |
| *Escherichia coli CE549* | 1 |
| *Escherichia coli chi7122* | 1 |
| *Escherichia coli D6-117.29* | 1 |
| *Escherichia coli DEC11A* | 1 |
| *Escherichia coli DEC11B* | 1 |
| *Escherichia coli DEC11E* | 1 |
| *Escherichia coli DEC12A* | 1 |
| *Escherichia coli DEC12B* | 1 |
| *Escherichia coli DEC12E* | 1 |
| *Escherichia coli DEC14A* | 1 |
| *Escherichia coli DEC14B* | 1 |
| *Escherichia coli DEC14D* | 1 |
| *Escherichia coli DEC7A* | 1 |
| *Escherichia coli DEC7C* | 1 |
| *Escherichia coli DEC7D* | 1 |
| *Escherichia coli DEC7E* | 1 |
| *Escherichia coli DH1* | 2 |
| *Escherichia coli E1002* | 1 |
| *Escherichia coli E110019* | 1 |
| *Escherichia coli E1114* | 1 |
| *Escherichia coli E1140* | 1 |
| *Escherichia coli E1520* | 1 |
| *Escherichia coli E1728* | 1 |
| *Escherichia coli E22* | 1 |
| *Escherichia coli E482* | 1 |
| *Escherichia coli E560* | 1 |
| *Escherichia coli E704* | 1 |
| *Escherichia coli ECA-727* | 1 |
| *Escherichia coli ECC-Z* | 1 |
| *Escherichia coli EPECa12* | 1 |
| *Escherichia coli ER2796* | 1 |
| *Escherichia coli ETEC H10407* | 1 |
| *Escherichia coli FAP1* | 1 |
| *Escherichia coli G3/10* | 1 |
| *Escherichia coli H299* | 1 |
| *Escherichia coli H386* | 1 |
| *Escherichia coli H420* | 1 |
| *Escherichia coli H454* | 1 |
| *Escherichia coli H489* | 1 |
| *Escherichia coli H494* | 1 |
| *Escherichia coli H617* | 1 |
| *Escherichia coli H730* | 1 |
| *Escherichia coli HS* | 2 |
| *Escherichia coli HVH 115 (4-4465989)* | 1 |
| *Escherichia coli HVH 115 (4-4465997)* | 1 |
| *Escherichia coli HVH 121 (4-6877826)* | 1 |
| *Escherichia coli HVH 139 (4-3192644)* | 1 |
| *Escherichia coli HVH 150 (4-3258106)* | 1 |
| *Escherichia coli HVH 152 (4-3447545)* | 1 |
| *Escherichia coli HVH 164 (4-5953081)* | 1 |
| *Escherichia coli HVH 18 (4-8589585)* | 1 |
| *Escherichia coli HVH 209 (4-3062651)* | 1 |
| *Escherichia coli HVH 221 (4-3136817)* | 1 |
| *Escherichia coli HVH 50 (4-2593475)* | 1 |
| *Escherichia coli HVH 79 (4-2512823)* | 1 |
| *Escherichia coli HVH 82 (4-2209276)* | 1 |
| *Escherichia coli HVH 91 (4-4638751)* | 1 |
| *Escherichia coli HVH 98 (4-5799287)* | 1 |
| *Escherichia coli IAI1* | 2 |
| *Escherichia coli KLY* | 1 |
| *Escherichia coli KO11FL* | 2 |
| *Escherichia coli KOEGE 131 (358a)* | 1 |
| *Escherichia coli KOEGE 33 (68a)* | 1 |
| *Escherichia coli KOEGE 40 (102a)* | 1 |
| *Escherichia coli KOEGE 7 (16a)* | 1 |
| *Escherichia coli KRX* | 1 |
| *Escherichia coli KTE10* | 1 |
| *Escherichia coli KTE100* | 1 |
| *Escherichia coli KTE101* | 1 |
| *Escherichia coli KTE103* | 1 |
| *Escherichia coli KTE107* | 1 |
| *Escherichia coli KTE108* | 1 |
| *Escherichia coli KTE111* | 1 |
| *Escherichia coli KTE119* | 1 |
| *Escherichia coli KTE12* | 1 |
| *Escherichia coli KTE120* | 1 |
| *Escherichia coli KTE13* | 1 |
| *Escherichia coli KTE130* | 1 |
| *Escherichia coli KTE132* | 1 |
| *Escherichia coli KTE135* | 1 |
| *Escherichia coli KTE14* | 1 |
| *Escherichia coli KTE142* | 1 |
| *Escherichia coli KTE154* | 1 |
| *Escherichia coli KTE163* | 1 |
| *Escherichia coli KTE184* | 1 |
| *Escherichia coli KTE197* | 1 |
| *Escherichia coli KTE198* | 1 |
| *Escherichia coli KTE210* | 1 |
| *Escherichia coli KTE212* | 1 |
| *Escherichia coli KTE221* | 1 |
| *Escherichia coli KTE232* | 1 |
| *Escherichia coli KTE233* | 1 |
| *Escherichia coli KTE234* | 1 |
| *Escherichia coli KTE29* | 1 |
| *Escherichia coli KTE34* | 1 |
| *Escherichia coli KTE35* | 1 |
| *Escherichia coli KTE40* | 1 |
| *Escherichia coli KTE41* | 1 |
| *Escherichia coli KTE44* | 1 |
| *Escherichia coli KTE48* | 1 |
| *Escherichia coli KTE51* | 1 |
| *Escherichia coli KTE56* | 1 |
| *Escherichia coli KTE61* | 1 |
| *Escherichia coli KTE73* | 1 |
| *Escherichia coli KTE77* | 1 |
| *Escherichia coli KTE90* | 1 |
| *Escherichia coli KTE91* | 1 |
| *Escherichia coli LY180* | 1 |
| *Escherichia coli MGH 57* | 1 |
| *Escherichia coli MP021017.1* | 1 |
| *Escherichia coli MP021017.10* | 1 |
| *Escherichia coli MP021017.12* | 1 |
| *Escherichia coli MP021017.4* | 1 |
| *Escherichia coli MP021017.6* | 1 |
| *Escherichia coli MP021017.9* | 1 |
| *Escherichia coli MP021552.11* | 1 |
| *Escherichia coli MP021552.12* | 1 |
| *Escherichia coli MP021552.8* | 1 |
| *Escherichia coli MP021566.1* | 1 |
| *Escherichia coli MS 116-1* | 1 |
| *Escherichia coli MS 175-1* | 1 |
| *Escherichia coli MS 187-1* | 1 |
| *Escherichia coli MS 196-1* | 1 |
| *Escherichia coli MS 78-1* | 1 |
| *Escherichia coli MS 84-1* | 1 |
| *Escherichia coli MS 85-1* | 1 |
| *Escherichia coli multispecies* | 1125 |
| *Escherichia coli N36254PS* | 1 |
| *Escherichia coli N36410PS* | 1 |
| *Escherichia coli N37058PS* | 1 |
| *Escherichia coli N37122PS* | 1 |
| *Escherichia coli N37139PS* | 1 |
| *Escherichia coli N40607* | 1 |
| *Escherichia coli NCCP15648* | 1 |
| *Escherichia coli NCTC 50110* | 1 |
| *Escherichia coli O08* | 1 |
| *Escherichia coli O10:K5(L):H4 str. ATCC 23506* | 1 |
| *Escherichia coli O103:H2 str. 12009* | 1 |
| *Escherichia coli O103:H2 str. 2010C-4433* | 1 |
| *Escherichia coli O103:H2 str. 2011C-3750* | 1 |
| *Escherichia coli O103:H2 str. CVM9450* | 1 |
| *Escherichia coli O103:H25 str. 2010C-4529* | 1 |
| *Escherichia coli O103:H25 str. CVM9340* | 1 |
| *Escherichia coli O104:H21 str. 94-3025* | 1 |
| *Escherichia coli O104:H21 str. CFSAN002236* | 1 |
| *Escherichia coli O104:H21 str. CFSAN002237* | 1 |
| *Escherichia coli O104:H4* | 1 |
| *Escherichia coli O104:H4 str. 01-09591* | 1 |
| *Escherichia coli O104:H4 str. 04-8351* | 1 |
| *Escherichia coli O104:H4 str. 09-7901* | 1 |
| *Escherichia coli O104:H4 str. 11-02030* | 1 |
| *Escherichia coli O104:H4 str. 11-02033-1* | 1 |
| *Escherichia coli O104:H4 str. 11-02092* | 1 |
| *Escherichia coli O104:H4 str. 11-02093* | 1 |
| *Escherichia coli O104:H4 str. 11-02281* | 1 |
| *Escherichia coli O104:H4 str. 11-02318* | 1 |
| *Escherichia coli O104:H4 str. 11-02913* | 1 |
| *Escherichia coli O104:H4 str. 11-03439* | 1 |
| *Escherichia coli O104:H4 str. 11-03943* | 1 |
| *Escherichia coli O104:H4 str. 11-04080* | 1 |
| *Escherichia coli O104:H4 str. 11-3677* | 1 |
| *Escherichia coli O104:H4 str. 11-4404* | 1 |
| *Escherichia coli O104:H4 str. 11-4522* | 1 |
| *Escherichia coli O104:H4 str. 11-4623* | 1 |
| *Escherichia coli O104:H4 str. 11-4632 C1* | 1 |
| *Escherichia coli O104:H4 str. 11-4632 C2* | 1 |
| *Escherichia coli O104:H4 str. 11-4632 C3* | 1 |
| *Escherichia coli O104:H4 str. 11-4632 C4* | 1 |
| *Escherichia coli O104:H4 str. 11-4632 C5* | 1 |
| *Escherichia coli O104:H4 str. 2009EL-2050* | 1 |
| *Escherichia coli O104:H4 str. 2009EL-2071* | 1 |
| *Escherichia coli O104:H4 str. 2011C-3493* | 2 |
| *Escherichia coli O104:H4 str. 2011EL-1675A* | 1 |
| *Escherichia coli O104:H4 str. C227-11* | 3 |
| *Escherichia coli O104:H4 str. C236-11* | 1 |
| *Escherichia coli O104:H4 str. E112/10* | 1 |
| *Escherichia coli O104:H4 str. E92/11* | 1 |
| *Escherichia coli O104:H4 str. Ec11-4984* | 1 |
| *Escherichia coli O104:H4 str. Ec11-4986* | 1 |
| *Escherichia coli O104:H4 str. Ec11-4987* | 1 |
| *Escherichia coli O104:H4 str. Ec11-4988* | 1 |
| *Escherichia coli O104:H4 str. Ec11-5603* | 1 |
| *Escherichia coli O104:H4 str. Ec11-5604* | 1 |
| *Escherichia coli O104:H4 str. Ec11-6006* | 1 |
| *Escherichia coli O104:H4 str. Ec11-9450* | 1 |
| *Escherichia coli O104:H4 str. Ec11-9941* | 1 |
| *Escherichia coli O104:H4 str. Ec11-9990* | 1 |
| *Escherichia coli O104:H4 str. Ec12-0465* | 1 |
| *Escherichia coli O104:H4 str. Ec12-0466* | 1 |
| *Escherichia coli O104:H4 str. LB226692* | 1 |
| *Escherichia coli O121:H19* | 1 |
| *Escherichia coli O121:H19 str. 03-3227* | 1 |
| *Escherichia coli O121:H19 str. 06-3003* | 1 |
| *Escherichia coli O121:H19 str. 06-3822* | 1 |
| *Escherichia coli O121:H19 str. 2009C-4050* | 1 |
| *Escherichia coli O121:H19 str. 2009C-4659* | 1 |
| *Escherichia coli O121:H19 str. 2009C-4750* | 1 |
| *Escherichia coli O121:H19 str. 2009EL1302* | 1 |
| *Escherichia coli O121:H19 str. 2009EL1412* | 1 |
| *Escherichia coli O121:H19 str. 2010C-3609* | 1 |
| *Escherichia coli O121:H19 str. 2010C-3794* | 1 |
| *Escherichia coli O121:H19 str. 2010C-3840* | 1 |
| *Escherichia coli O121:H19 str. 2010C-4254* | 1 |
| *Escherichia coli O121:H19 str. 2010C-4732* | 1 |
| *Escherichia coli O121:H19 str. 2010C-4824* | 1 |
| *Escherichia coli O121:H19 str. 2010C-4966* | 1 |
| *Escherichia coli O121:H19 str. 2010C-4989* | 1 |
| *Escherichia coli O121:H19 str. 2010EL1058* | 1 |
| *Escherichia coli O121:H19 str. 2011C-3072* | 1 |
| *Escherichia coli O121:H19 str. 2011C-3108* | 1 |
| *Escherichia coli O121:H19 str. 2011C-3216* | 1 |
| *Escherichia coli O121:H19 str. 2011C-3500* | 1 |
| *Escherichia coli O121:H19 str. 2011C-3537* | 1 |
| *Escherichia coli O121:H19 str. 2011C-3609* | 1 |
| *Escherichia coli O121:H19 str. F6714* | 1 |
| *Escherichia coli O121:H19 str. K5198* | 1 |
| *Escherichia coli O121:H19 str. K5269* | 1 |
| *Escherichia coli O123:H11 str. 2009C-3307* | 1 |
| *Escherichia coli O128:H2 str. 2011C-3317* | 1 |
| *Escherichia coli O139:H28 str. E24377A* | 1 |
| *Escherichia coli O145:H28 str. 4865/96* | 1 |
| *Escherichia coli O145:H28 str. RM12581* | 1 |
| *Escherichia coli O145:H28 str. RM12761* | 1 |
| *Escherichia coli O145:H28 str. RM13514* | 1 |
| *Escherichia coli O145:H28 str. RM13516* | 1 |
| *Escherichia coli O145:NM str. 06-3484* | 1 |
| *Escherichia coli O145:NM str. 08-4270* | 1 |
| *Escherichia coli O145:NM str. 2010C-3507* | 1 |
| *Escherichia coli O145:NM str. 2010C-3508* | 1 |
| *Escherichia coli O145:NM str. 2010C-3509* | 1 |
| *Escherichia coli O145:NM str. 2010C-3510* | 1 |
| *Escherichia coli O145:NM str. 2010C-3511* | 1 |
| *Escherichia coli O145:NM str. 2010C-3516* | 1 |
| *Escherichia coli O145:NM str. 2010C-3517* | 1 |
| *Escherichia coli O145:NM str. 2010C-3518* | 1 |
| *Escherichia coli O145:NM str. 2010C-3521* | 1 |
| *Escherichia coli O145:NM str. 2010C-3526* | 1 |
| *Escherichia coli O145:NM str. 2010C-4557C2* | 1 |
| *Escherichia coli O146:H21 str. 2010C-3325* | 1 |
| *Escherichia coli O153:H2 str. 2010C-5034* | 1 |
| *Escherichia coli O156:H25 str. 2011C-3602* | 1 |
| *Escherichia coli O174:H21 str. 03-3269* | 1 |
| *Escherichia coli O174:H8 str. 04-3038* | 1 |
| *Escherichia coli O25:NM str. E2539C1* | 1 |
| *Escherichia coli O32:H37 str. P4* | 1 |
| *Escherichia coli O39:NM str. F8704-2* | 1 |
| *Escherichia coli O45:H2 str. 01-3147* | 1 |
| *Escherichia coli O45:H2 str. 2009C-3686* | 1 |
| *Escherichia coli O45:H2 str. 2009C-4780* | 1 |
| *Escherichia coli O45:H2 str. 2010C-3876* | 1 |
| *Escherichia coli O45:H2 str. 2010C-4211* | 1 |
| *Escherichia coli O5:K4(L):H4 str. ATCC 23502* | 1 |
| *Escherichia coli O6:H16 str. 99-3165* | 1 |
| *Escherichia coli O6:H16 str. F5656C1* | 1 |
| *Escherichia coli O6:H16:CFA/II str. B2C* | 1 |
| *Escherichia coli O91 str. RM7190* | 1 |
| *Escherichia coli O91:H21 str. 2009C-3740* | 1 |
| *Escherichia coli O91:H21 str. 2009C-4646* | 1 |
| *Escherichia coli P0299438.10* | 1 |
| *Escherichia coli P0299438.4* | 1 |
| *Escherichia coli P0299438.9* | 1 |
| *Escherichia coli P0299483.2* | 1 |
| *Escherichia coli P0299917.1* | 1 |
| *Escherichia coli P0301904.3* | 1 |
| *Escherichia coli P0304816.12* | 1 |
| *Escherichia coli P0304816.7* | 1 |
| *Escherichia coli P0305260.1* | 1 |
| *Escherichia coli P0305260.10* | 1 |
| *Escherichia coli P0305260.11* | 1 |
| *Escherichia coli P0305260.12* | 1 |
| *Escherichia coli P0305260.15* | 1 |
| *Escherichia coli P0305260.2* | 1 |
| *Escherichia coli P0305260.5* | 1 |
| *Escherichia coli P0305260.9* | 1 |
| *Escherichia coli P12b* | 1 |
| *Escherichia coli P4-96* | 1 |
| *Escherichia coli P4-NR* | 1 |
| *Escherichia coli PCN009* | 1 |
| *Escherichia coli PCN061* | 1 |
| *Escherichia coli S17* | 1 |
| *Escherichia coli SE11* | 2 |
| *Escherichia coli SEPT362* | 1 |
| *Escherichia coli STEC_94C* | 1 |
| *Escherichia coli STEC_O31* | 1 |
| *Escherichia coli str. K-12 substr. DH10B* | 2 |
| *Escherichia coli str. K-12 substr. MC4100* | 1 |
| *Escherichia coli str. K-12 substr. MDS42* | 1 |
| *Escherichia coli str. K-12 substr. MG1655* | 7 |
| *Escherichia coli str. K-12 substr. W3110* | 2 |
| *Escherichia coli TA007* | 1 |
| *Escherichia coli TA008* | 1 |
| *Escherichia coli TA144* | 1 |
| *Escherichia coli ThroopD* | 1 |
| *Escherichia coli TW10598* | 1 |
| *Escherichia coli TX1999* | 1 |
| *Escherichia coli UCI 53* | 1 |
| *Escherichia coli UCI 66* | 1 |
| *Escherichia coli UMEA 3052-1* | 1 |
| *Escherichia coli UMEA 3065-1* | 1 |
| *Escherichia coli UMEA 3139-1* | 1 |
| *Escherichia coli UMEA 3148-1* | 1 |
| *Escherichia coli UMEA 3174-1* | 1 |
| *Escherichia coli UMEA 3176-1* | 1 |
| *Escherichia coli UMEA 3180-1* | 1 |
| *Escherichia coli UMEA 3199-1* | 1 |
| *Escherichia coli UMEA 3201-1* | 1 |
| *Escherichia coli UMEA 3212-1* | 1 |
| *Escherichia coli UMEA 3240-1* | 1 |
| *Escherichia coli UMEA 3271-1* | 1 |
| *Escherichia coli UMEA 3292-1* | 1 |
| *Escherichia coli UMEA 3317-1* | 1 |
| *Escherichia coli UMEA 3318-1* | 1 |
| *Escherichia coli UMEA 3329-1* | 1 |
| *Escherichia coli UMEA 3336-1* | 1 |
| *Escherichia coli UMEA 3682-1* | 1 |
| *Escherichia coli UMNK88* | 1 |
| *Escherichia coli VL2732* | 1 |
| *Escherichia coli VR50* | 1 |
| *Escherichia coli W* | 3 |
| *Escherichia coli XH001* | 1 |
| *Escherichia coli XH140A* | 1 |
| synthetic *Escherichia coli C321.deltaA* | 1 |

Supplementary Table S4. Selected Reaction Monitoring (SRM) parameters for LC-MS detection of compounds and cross-contamination.

| Compound | Retention time (min) | Precursor (m/z) | Product (m/z) | Collision Energy (V) |
| --- | --- | --- | --- | --- |
| B1 | 1.5 | 265.16 | 265.16 | 5 |
| B1 | 1.5 | 265.16 | 144.2 | 15.8 |
| B1 | 1.5 | 265.16 | 122.27 | 16.2 |
| B1 | 1.5 | 265.16 | 81.25 | 29.3 |
| B1 | 1.5 | 265.16 | 42.354 | 37.3 |
| cHET | 6.3 | 188.07 | 188.07 | 5 |
| cHET | 6.3 | 188.07 | 170.13 | 14.6 |
| cHET | 6.3 | 188.07 | 152.17 | 22 |
| cHET | 6.3 | 188.07 | 140.17 | 21.1 |
| cHET | 6.3 | 188.07 | 111.18 | 30.2 |
| cHET | 6.3 | 188.07 | 70.25 | 36.5 |
| cHET | 6.3 | 188.07 | 85.23 | 26.9 |
| HET | 3.9 | 144.07 | 144.07 | 5 |
| HET | 3.9 | 144.07 | 126.23 | 19.5 |
| HET | 3.9 | 144.07 | 113.2 | 24 |
| HET | 3.9 | 144.07 | 112.2 | 32 |
| HET | 3.9 | 144.07 | 99.23 | 21.9 |
| HET | 3.9 | 144.07 | 80.25 | 32 |
| HET | 3.9 | 144.07 | 71.3 | 35.8 |
| HMP | 1.6 | 140.1 | 140.1 | 5 |
| HMP | 1.6 | 140.1 | 122.2 | 12.3 |
| HMP | 1.6 | 140.1 | 81.3 | 19.5 |
| HMP | 1.6 | 140.1 | 54.3 | 18.7 |
